# A Case of Congenital Heart-Disease

**Published:** 1890-09

**Authors:** Lockhart Stephens

**Affiliations:** Emsworth


### A CASE OF CONGENITAL HEART-DISEASE.

By Lockhart Stephens, M.R.C.S., L.S.A., Emsworth.

As it is desirable to have a permanent record of any considerable deviation from normal anatomical formation, the following notes will be of interest.

M. L., æt. 2 years and 4 months, was admitted on May 26th, 1885, to the Bristol General Hospital under the care of Mr. Pickering, to whom I am indebted for permission to publish the case, for severe burn of buttocks and lower part of back, which, although treated in the usual manner, showed little tendency to heal. The child was extremely livid,* even to the finger-tips, which were markedly clubbed. It was a poor, ill-nourished child; at times bright and cheerful, at others troubled a good deal with shortness of breath. The only condition to

* The lividity increased considerably during stay in Hospital.

be made out during life was that the apex-beat was in the usual situation; there was a systolic thrill and bruit. Much importance, however, cannot be attached to this observation; for the child was too ill to admit of thorough examination. The blueness had existed since birth, according to the mother's account.

June 15th.—Had a convulsive attack and nearly died.

June 16th.—More blue than usual. Died suddenly, after convulsion.

Examination twenty-four hours after death.

Body thin and ill-nourished. A large granulating surface, 4 × 3 in., over sacrum and upper part of buttocks. No signs of decomposition. Day warm and dry. Rigor mortis slight. No hypostasis.

Nothing unusual about external appearance of body beyond the extreme clubbing of the nails.

Head.—Not examined.

Neck.—No enlarged glands. Nothing abnormal about thymus, thyroid, ribs, or costal cartilages.

Lungs and Pleuræ.—Healthy. Were removed entire, with heart and liver, in order to see the arrangement of the great vessels.

Heart.—Not weighed, but larger than normal. On opening pericardium ʒij. or ʒiij. of clear yellow serum found in sac. Heart very firm and rounded; this condition being almost entirely due to the right ventricle, which was firm and tense, and formed the apex of the organ. The aorta was found to arise from the right ventricle and the pulmonary artery from the left ventricle.

To summarise the arrangement existing between the cavities of the heart and the great vessels, it was as follows:

I. *Right Auricle.*—Very large and filled with clot.

Opening into it from below was the vena cava inferior, the size of one's forefinger; from above the vena cava superior, the size of a quill-pen or rather larger.

Between the right and left auricles is the foramen ovale, showing a firm crescentic margin for three-fourths of its circumference, whilst the posterior, or rather postero-inferior, quarter blended with the muscular wall of the auricle, towards the orifice of entrance of the inferior cava. The valve is fenestrated in five small holes around the quarter of its circumference nearest the inferior cava and just in front of the orifice of entrance of the right pulmonary vein to the left auricle.

A probe passes directly from right to left auricles, from inferior cava to left auricle, and from right auricle through the valve to the right pulmonary vein. The coronary sinus also opens into right auricle.

II. *Right Ventricle.*—Very thick, half inch at thickest part near apex, and strong. The aorta comes off from it in front of the pulmonary artery, which comes from the left ventricle. The aortic and auriculo-ventricular valves are healthy.

III. *Left Ventricle.*—Thin, one-tenth inch thick. From it the pulmonary artery arises and into it the left auricle opens. The valves of both orifices are healthy.

IV. *Left Auricle.*—Very minute; just a little puckered piece of heart-muscle and no more.

Where the right and left pulmonary veins meet, the anterior wall of the vessel is made up of the fenestrated foramen ovale. There is practically no left auricle.

The ductus arteriosus is closed.

